# A systematic review of urine biomarkers in children with IgA vasculitis nephritis

**DOI:** 10.1007/s00467-021-05107-7

**Published:** 2021-05-15

**Authors:** Chloe E. C. Williams, Aileen Toner, Rachael D. Wright, Louise Oni

**Affiliations:** 1 School of Medicine, University of Liverpool, Liverpool, UK; 2 Department of Women’s and Children’s Health, Institute of Life Course and Medical Sciences, University of Liverpool, Liverpool, UK; 3 Department of Paediatric Nephrology, Alder Hey Children’s NHS Foundation Trust Hospital, Eaton Road, Liverpool, L12 2AP UK

**Keywords:** IgA vasculitis, Henoch-Schönlein purpura, Nephritis, Children, Urine, Biomarker

## Abstract

**Background:**

Nephritis is a recognised complication of IgA vasculitis (IgAV, Henoch-Schönlein purpura) contributing to 1–2% of all chronic kidney disease (CKD) stage 5. Improved understanding may reduce irreversible damage in IgAV nephritis (IgAV-N).

**Objective:**

The aim of this study was to perform a comprehensive systematic literature review to identify promising clinical and pre-clinical urine biomarkers in children with IgAV-N that could predict the presence of nephritis and/or determine its severity.

**Methods:**

A systematic literature review was performed using four search engines and a predefined search term strategy. Promising biomarkers were divided in terms of clinical or pre-clinical and ability to predict the presence of nephritis or determine its severity. Results were described using statistical significance (*p* < 0.05) and area under the curve (AUC) values.

**Results:**

One hundred twenty-one studies were identified; 13 were eligible. A total of 2446 paediatric patients were included: healthy controls (*n* = 761), children with IgAV-N (*n* = 1236) and children with IgAV without nephritis (IgAV-noN, *n* = 449). Fifty-one percent were male, median age 7.9 years. The clinical markers, 24-h protein quantity and urine protein:creatinine ratio, were deemed acceptable for assessing severity of nephritis (AUC < 0.8). Urinary albumin concentration (Malb) performed well (AUC 0.81–0.98). The most promising pre-clinical urinary biomarkers in predicting presence of nephritis were as follows: kidney injury molecule-1 (KIM-1) (AUC 0.93), monocyte chemotactic protein-1 (MCP-1) (AUC 0.83), N-acetyl-β-glucosaminidase (NAG) (0.76–0.96), and angiotensinogen (AGT) (AUC not available). Urinary KIM-1, MCP-1, and NAG appeared to correlate with disease severity.

**Conclusions:**

Longitudinal studies are needed to assess whether pre-clinical biomarkers enhance standard of care in IgAV-N.

## Introduction

Immunoglobulin A (IgA) vasculitis (IgAV), formerly known as Henoch-Schönlein purpura (HSP), is the most common form of vasculitis in children, with an estimated incidence of 20.4 cases/100,000 childhood population [1, 2]. This systemic small vessel vasculitis usually presents with a palpable purpuric rash, plus polyarthritis, gastrointestinal (GI) symptoms and/or kidney involvement, and it is predominantly a disease of childhood. The exact pathophysiology is still unknown, but due to the high levels of galactose deficient IgA1 levels seen in IgAV patients, it is thought that aberrant IgA glycosylation is a contributor to the mechanism of disease. Immune complexes containing IgA1 then deposit in the small vessels activating an immune response and subsequent inflammation [3]. The prognosis of IgAV is usually excellent with 94% of children achieving full, spontaneous recovery within 2 years [4]. Around 40–50% of patients experience kidney inflammation (termed IgAV nephritis; IgAV-N) ranging from microscopic haematuria to rapidly progressive glomerulonephritis [5, 6] and it currently contributes to 1–2% of all chronic kidney disease (CKD) stage 5 [7]. For this reason, all patients should have a period of follow-up to screen for IgAV-N that currently consists of 6 months of periodic urinalysis and blood pressure monitoring, as surrogate clinical markers of kidney injury [8]. Identifying those individuals at greatest risk of kidney inflammation is believed to be the key to reducing the incidence of irreversible kidney damage in IgAV-N and allowing a personalised approach to monitoring. Pre-clinical biomarkers may have a role in identifying patients with or without nephritis and determining the severity of kidney inflammation. Ideally, to fulfil this role they should be reflective of the pathogenic biological process and be accurate and reproducible. For IgAV-N, this may provide earlier diagnosis of kidney inflammation, prognostic information, and scientific insight and ultimately allow personalised disease monitoring to stratify the management of children with this disease.

The primary aim of this study was to perform a comprehensive systematic literature review to identify promising clinical and pre-clinical urine biomarkers in children with IgAV that can either predict the presence of nephritis and/or determine its severity.

## Methods

### Study population

The inclusion criteria were paediatric participants (<18 years) of any sex and ethnicity, with a diagnosis of IgAV-N. A diagnosis of IgAV-N included any of the following: abnormal urinalysis; haematuria and/or a high urinary protein concentration within 6 months of the onset of rash; and/or a reduced estimated glomerular filtration rate (eGFR) in participants who had met the clinical diagnosis of IgAV [9]. The exclusion criteria were studies that involved adult participants (>18 years) or participants who had other forms of nephritis or vasculitis.

### Intervention

The intervention of interest was biomarker assay evaluation in a urine sample.

### Comparator

The study aimed to compare: (i) urine biomarkers that may determine the presence of nephritis in children with IgAV-N compared to children with IgAV and no nephritis (IgAV-noN) and/or healthy paediatric controls and (ii) urine biomarkers that may determine the severity of nephritis in children with IgAV-N.

### Outcome

The outcome of interest was the identification of clinical or pre-clinical biomarkers that are able to determine the presence of nephritis as defined by each individual study and/or the severity defined in terms of the International Study of Kidney Disease in Children (ISKDC) classification histological grade or extent of proteinuria [10].

### Study design

#### Data extraction

Using predefined methodology, this systematic review evaluated the current available literature. Four online databases, PubMed, Web of Science, Medline, and Scopus, were used with the following terms: (((((((((neonat*) OR (adolescen*)) OR (infan*)) OR (child*)) OR (pediatric*)) OR (paediatric*)) AND ((((((immunoglobulin A vasculitis) OR (IgA Vasculitis)) OR (IgAV)) OR (Henoch Sch*nlein purpura)) OR (Henoch-Sch*nlein purpura)) OR (HSP))) AND (((((((nephritis) OR (renal injur*)) OR (kidney injur*)) OR (renal damage*)) OR (kidney damage)) OR (ckd)) OR (chronic kidney disease))) AND (urin*)) AND (biomarker*). The studies included were meta-analyses, randomised control trials (RCTs), cohort studies, case-control studies, cross-sectional studies and case series (*n* > 5) that were all accessible in full text through the University of Liverpool, with at least an English abstract. Secondary data and animal studies were excluded, as well as papers with an original publication date before October 2000, allowing for a 20-year inclusion period. The reference lists of relevant literature were hand-searched to identify any additional eligible studies.

#### Data collection

From each included study, information was extracted on author, year of publication, study design, study population, definition of nephritis, type of sampling and laboratory technique, biomarkers assessed, and key findings. The relevant data was collected on a predesigned pro forma by the primary author (CW). Where full English transcripts were unavailable, data was extracted from the English abstract.

### Quality appraisal and statistical analysis

The “Appraisal tool for Cross-Sectional Studies” (AXIS) tool was used, which comprises 20 questions to appraise and compare the quality of the literature [11]. Pre-clinical biomarkers identified in more than one paper were to be discussed in more detail. Those that have only been reported once were to be summarised in a data table (Table 1). The results will be described in terms of clinical or pre-clinical biomarkers. A clinical biomarker is defined as any biological marker that is available in a routine clinical laboratory. A pre-clinical biomarker is one that is not routinely available in a clinical laboratory and deemed experimental [25]. Where available, descriptive statistics will be presented as percentage male and a median age will be calculated using the available age data. Laboratory data will be presented as either a mean with standard deviation or as a median with range depending on the original publication. Area under the curve (AUC) will be presented to represent the strength of the biomarker and described as a value from 0–1.0 with a 95% confidence interval. In terms of biomarker strength, an AUC of ≤ 0.5 suggests no discrimination, 0.7–0.8 is considered acceptable, 0.8–0.9 is considered excellent, and ≥ 0.9 is considered outstanding [26]. *p*-values < 0.05 and a confidence interval which does not overlap 0 will be considered significant. As it was expected that the studies revealed would be heterogeneous, a meta-analysis was not conducted.

**Table 1:** A table describing the data in each paper included in the systematic review

Author	Year	Study design	Cohort demographic	Definition of nephritis	Type of sampling	Laboratory technique	Biomarker	Results
An and Xia [12]	2018	Retrospective cross sectional	45 children with biopsy-confirmed IgAV-N grouped by pathological grade.	Kidney histology, classified according to ISKDC.	24-h urine collection	Turbidimetric method	Beta-2 microglobulin (β2-MG) Urinary albumin concentration (Malb) N-Acetyl-beta-glucosaminidase (NAG) Transferrin (TfR)	Malb, TfR and NAG were different according to pathological grades (*p* < 0.05). β2-MG was not statistically significantly increased.
Dyga et al. [13]	2020	Prospective longitudinal	11 paediatric patients IgAV-N (M = 10, F = 1) and 18 with IgAV-noN (M = 7, F = 11) compared to 34 healthy controls (M = 23, F = 11).	Haematuria: >5 erythrocytes per high power field ± UP/UC ratio > 30 mg/mmol ± eGFR < 60 mL/min/1.73 m^2^.	One acute random urine sample and follow-up sample 2–6 months after discharge	ELISA	Neutrophil gelatinase-associated lipocalin (NGAL) Kidney injury molecule-1 (KIM-1) Liver-fatty acid binding protein (L-FABP)	Acutely, all three biomarkers were increased in children with IgAV compared to controls (*p* < 0.001), however, not between the IgAV-N and IgAV-noN groups. At follow-up, NGAL was found to be increased in IgAV-N compared to IgAV-noN (*p* = 0.063).
Fang et al. [14]	2020	Prospective cross sectional	30 children with IgAV-N (M = 20, F = 10) compared to 10 IgAV-noN (M = 6, F = 4) and 29 healthy controls (M = 12, F = 17).	Haematuria and/or high urinary protein concentration or kidney biopsy results showing mesangial IgA deposition.	Midstream morning urine sample	ELISA	Integrin beta-1 (ITGB1) Tenascin	There were decreased urinary concentrations of both biomarkers in the IgAV-N cohort compared to controls (*p* < 0.05). Tenascin was statistically significantly different in the IgAV-N vs. IgAV-noN (*p* = 0.005).
Fuentes et al. [15]	2014	Prospective cross sectional	57 children had IgAV-N (M = 32, F = 25) and 20 with IgAV-noN (M = 12, F = 8), compared to 25 healthy volunteers (M = 16, F = 9).	Haematuria (>5 cells per high-power field in urine sediment) and/or high urinary protein concentration. Kidney biopsy was classified using the ISKDC criteria.	First-morning urine sample	ELISA	Monocyte chemoattractant protein-1 (MCP-1)	Urinary MCP-1/Cr was increased in IgAV-N compared to the IgAV-noN and the controls (*p* < 0.0001).
Ge et al. [16]	2014	Prospective longitudinal	34 paediatric patients with IgAV-noN (M = 15, F = 18), 37 with IgAV-N (M = 18, F = 19) and 37 healthy children (M = 19, F = 18).	Haematuria and/or high urinary protein concentration.	24-h urine collection	ELISA	Urinary albumin concentration (Malb) Beta-2 microglobulin (β2-MG)	The concentrations were increased in IgAV-N patients compared to controls (*p* < 0.05) and IgAV-noN (*p* < 0.05).
Ma et al. [17]	2020	Prospective longitudinal	14 children with IgAV-N (M = 7, F = 7) vs. 28 with IgAV-noN (M = 16, F = 12) and 23 healthy volunteers (M = 9, F = 14).	N/A^a^	Morning urine sample	N/A^a^	Urinary angiotensinogen (UAGT) Fibroblast specific protein-1 (FSP-1) Thrombin	UAGT and FSP-1 were increased in the IgAV-N cohort compared to controls and IgAV-noN (*p* < 0.05). Thrombin was increased in all IgAV patients when compared to controls (*p* < 0.05).
Mao et al. [18]	2012	Prospective longitudinal	51 paediatric patients with IgAV-noN (M = 24, F = 27) compared to 43 with haematuria but a urinary protein concentration of 0 (M = 21, F = 22) and 13 with high urinary protein concentration (M = 5, F = 8).	Urinary protein concentration (>1.0 g/24 h) and/or haematuria.	24-h urine sample collected acutely and at follow-up	ELISA	Urinary angiotensinogen (UAGT)	Acutely, UAGT concentrations were higher in those with a higher urinary protein concentration compared to IgAV-noN and IgAV with haematuria groups (*p* < 0.0001). During the convalescent phase, UAGT concentrations were increased in the patients with high urinary protein concentration compared to IgAV-noN patients (*p* < 0.0001) and the haematuria group (*p* < 0.001).
Pillebout et al. [19]	2017	Prospective cross sectional	21 paediatric controls (M = 13, F = 8) were compared to 17 children with IgAV-noN (M = 12, F = 5) and 33 children with IgAV-N (M = 20, F = 13).	The presence of haematuria and/or a PCR > 0.5 g/g and/or an eGFR < 60 mL/min/1.73 m^2^.	N/A^b^	ELISA	IgA/Cr ratio (IgA/Cr) IgG/Cr ratio (IgG/Cr) IgM/Cr ratio (IgM/Cr) Igλ/IgΚ ratio (Igλ/IgΚ) IL-6/Cr ratio (IL-6/Cr) IL-8/Cr ratio (IL-8/Cr) IL-10/Cr ratio (IL10/Cr)	IgA/Cr and IgM/Cr were raised in IgAV-N compared to both controls and IgAV-noN (*p* < 0.0001). IgG/Cr and the Igλ/IgΚ ratios were increased in IgAV-N compared to IgAV-noN (*p* < 0.01). IL-6/Cr and IL-8/Cr were increased in IgAV-N compared to controls (*p* < 0.0001) and IgAV-noN (*p* < 0.01). IL-2/Cr was increased only when compared to IgAV-noN (*p* < 0.01).
Qin et al. [20]	2011	Prospective cross sectional	68 children with IgAV-noN (M = 33, F = 35) were compared to 66 with IgAV-N (M = 32, F = 34) and 60 controls (M = 29, F = 31).	Patients categorised into normal concentrations of protein and haematuria; low-grade urinary protein concentration (< 1 g/L) and/or haematuria; and high urinary protein concentration (≥1 g/L) and/or haematuria.	Mid-stream urine sample	ELISA	Matrix metalloproteinase-9 (MMP-9) Tissue inhibitor matrix metalloproteinase-1 (TIMP-1)	Urinary MMP-9, TIMP-1 and MMP-9/TIMP-1 were increased in IgAV-N compared to IgAV-noN (*p* < 0.05) and controls (*p* < 0.01). MMP-9 and MMP-9/TIMP-1 were increased in children with high urinary protein concentration compared to mild (*p* < 0.01) and moderate (*p* < 0.05).
Wang et al. [21]	2017	Prospective cross sectional	126 paediatric patients with IgAV-N (M = 66, F = 60) were compared to 135 non-nephritis IgAV children (M = 71, F = 64) and 84 healthy controls (M = 48, F = 36).	Haematuria and/or high urinary protein concentration within 6 months of the onset of rash. IgAV-N patients were further grouped into mild/moderate/severely high urinary protein concentration.	First-morning urine sample	ELISA	Monocyte chemoattractant protein-1 (MCP-1)	Urinary MCP-1 was increased in IgAV-N compared to controls and IgAV-noN (*p* < 0.001). Concentrations also increased in parallel with the degree of urinary protein concentration (all *p* < 0.01).
Wang et al. [22]	2017	Prospective longitudinal	35 children (M = 18, F = 17) with IgAV-N, 41 paediatric patients (M = 18, F = 23) with a diagnosis of IgAV-noN and 32 healthy controls (M = 17, F = 15).	Haematuria and/or high urinary protein concentration within 6 months after the onset of rash.	Midstream first morning urine sample before and after treatment	ELISA	Macrophage migration inhibitory factor (MIF)	Urinary MIF was greatest in group I and higher than group II or controls (both *p* < 0.05).
Ye et al. [23]	2015	Prospective cross sectional	694 children (M = 332, F = 362) with biopsy-proven IgAV-N, compared to 400 healthy controls (M = 188, F = 212).	Nephritis was graded according to the KDIGO criteria. Biopsy was classified according the ISKDC criteria.	N/A^b^	Roche Modular P800 biochemical analyser	24-h urinary protein (24h-UPRO) Urinary protein:Cr ratio (U-PCR)	There was an increase in 24-UPRO and U-PCR when comparing those with grades I or IIa to grades IIb, IIIa or IIIb (*p* < 0.01). 24-UPRO was increased in IgAV-N compared to controls (*p* < 0.01).
Zhang et al. [24]	2015	Prospective longitudinal	27 children with IgAV-noN (M = 19, F = 8) were compared to 32 paediatric patients with IgAV-N (M = 18, F = 14) and 16 healthy volunteers (M = 9, F = 7).	Those who underwent a kidney biopsy were graded according to ISKDC criteria.^c^	Spot morning urine samples	ELISA	Kidney injury molecule-1 (KIM-1) N-Acetyl-beta-glucosaminidase (NAG) Beta-2 microglobulin (β2-MG)	Urinary KIM-1 concentrations were increased in IgAV-N compared to IgAV and controls (*p* < 0.05). Patients with IgAV had an increased concentration of urinary KIM-1 compared to controls (*p* < 0.001). NAG was highest in IgAV-N (*p* < 0.05).

Abbreviations: *Cr*, creatinine; *eGFR*, estimated glomerular filtration rate; *ELISA*, enzyme-linked immunosorbent assay; *Ig*, immunoglobulin; *IgAV*, immunoglobulin A vasculitis; *IgAV-N*, immunoglobulin A vasculitis nephritis; *IgAV-noN*, immunoglobulin A vasculitis without nephritis; *IL*, interleukin; *ISKDC*, International Study of Kidney Disease in Children; *KDIGO*, Kidney Disease Improving Global Outcomes; *PCR*, protein:creatinine ratio; *UC*, urinary creatinine; *UP*, urinary protein

^a^As this study was not published in English, data was only extracted from the abstract and this information was not available

^b^Method of urine sampling was not specified

^c^Nephritis was not defined in this study

### Ethical approval

Ethical approval was not necessary for the performance of this review, as per the National Health Service Research Authority, as it involved secondary review of existing literature.

## Results

### Data extraction

**Fig. 1:**
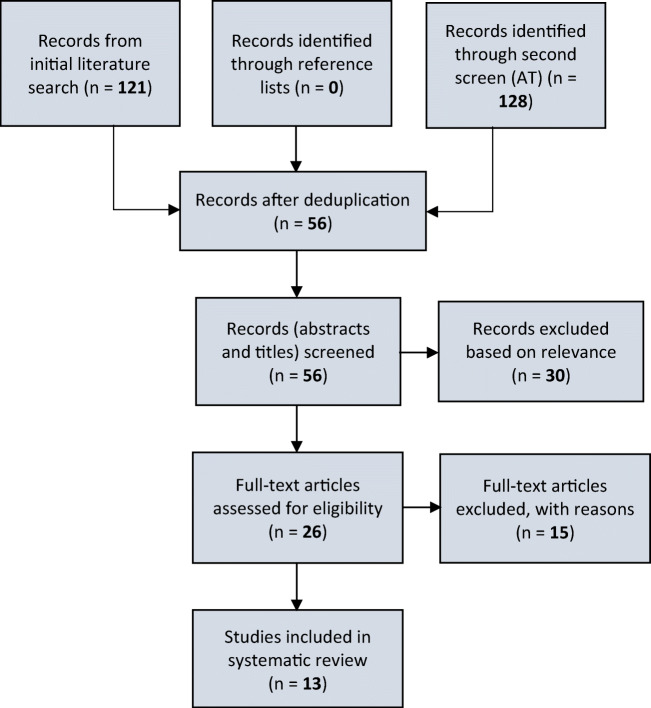
A flow diagram to represent the search and screen process. The systematic literature search was performed on 4 databases and returned 121 papers. Fifty-six papers were identified after deduplication. After screening by initial and a second independent person, a total of 13 studies were included in the systematic review

The search took place in September 2020 and yielded 121 papers. A total of 65 duplicates were removed leaving 56 titles eligible for abstract screening. Of these, 26 papers were eligible for full text review. After full text review, 11 were included in the systematic review. A second, independent reviewer (AT) repeated the search, at a time point 1 month later, to identify papers and determined whether the studies met the inclusion criteria; 128 papers were retrieved and after deduplication, two additional papers were identified that met the inclusion criteria, producing a total of 13 papers (Fig. 1). No further eligible papers were discovered in searching the reference lists.

### Participants

A total cohort of 2446 children were included in this systematic review from 13 studies. The median age of the entire cohort was 7.9 years and 51% were male. Data on sex was not available in one study [12]. Median or mean age was not available in two papers [12, 15] and age ranges could not be calculated due to the heterogeneity of the papers in presenting demographic data.

The participants comprised 1236 children with IgAV-N (48% male, median age 8.0 years), 761 healthy paediatric controls (52% male, median age 7.9 years) and 449 children with IgAV-noN (52% male, median age 7.0 years). The publication dates spanned from 2011–2020 [13, 14, 17, 27] and included both longitudinal [13, 17, 18, 24, 28, 29] and cross-sectional studies [12, 14, 15, 19, 22, 23, 27]. The majority of the papers were published from China [12, 14, 17, 18, 22–24, 27, 28, 30], and three studies were from Poland [13], France [19] and Mexico [15].

### Quality appraisal

The quality appraisal produced a good median AXIS score of 16/20 (range 14–17). One study was excluded from the quality assessment as it was not available in full text in English and there was insufficient detail in the abstract [17]. Those studies with lower AXIS scores were mostly due to small sample size, single site recruitment, and no mention of study limitations.

### Identified biomarkers

A total of 23 urine biomarkers were discovered that had been reported to be associated with IgAV-N; 20 were pre-clinical and 3 considered clinical biomarkers (Table 2). Increased urinary protein concentration was the only clinical urine biomarker identified and had been measured using 24-h urinary protein (24h-UPRO) values, urinary protein:creatinine ratio (U-PCR) and urinary albumin concentration (Malb). There were 5 pre-clinical urine biomarkers that had been reported more than once and thus described in more detail, these were as follows: beta-2 microglobulin (β2-MG), kidney injury molecule-1 (KIM-1), monocyte chemoattractant protein-1 (MCP-1), N-acetyl-β-glucosaminidase (NAG) and urinary angiotensinogen (UAGT).

**Table 2:** Frequency of biomarker identification in this systematic review

Biomarker identified	Studies
Beta-2 microglobulin (β2-MG)	An and Xia [12] Ge et al. [28] Qin et al. [27] Zhang et al. [24]
24-h urinary protein (24h-UPRO)	Ye et al. [23]
Fibroblast specific protein-1 (FSP-1)	Ma et al. [17]
Immunoglobulin λ/immunoglobulin Κ ratio (Igλ/IgΚ ratio)	Pillebout et al. [19]
Immunoglobulin A/Cr ratio (IgA/Cr)^a^	Pillebout et al. [19]
Immunoglobulin G/Cr ratio (IgG/Cr)^a^	Pillebout et al. [19]
Immunoglobulin M/Cr ratio (IgM/Cr)^a^	Pillebout et al. [19]
Interleukin-6/Cr ratio (IL-6/Cr)^a^	Pillebout et al. [19]
Interleukin-8/Cr ratio (IL-8/Cr)^a^	Pillebout et al. [19]
Interleukin-10/Cr ratio (IL10/Cr)^a^	Pillebout et al. [19]
Integrin beta-1 (ITGB1)	Fang et al. [14]
Kidney injury molecule-1 (KIM-1)	Dyga et al. [13] Zhang et al. [24]
Liver-fatty acid binding protein (L-FABP)	Dyga et al. [13]
Urinary albumin concentration (Malb)	An and Xia [12] Ge et al. [28]
Monocyte chemoattractant protein-1 (MCP-1)	Fuentes et al. [15] Wang et al. [22]
Macrophage migration inhibitory factor (MIF)	Wang et al. [29]
Matrix metalloproteinase-9 (MMP-9)	Qin et al. [27]
N-Acetyl-beta-glucosaminidase (NAG)	An and Xia [12] Zhang et al. [24]
Neutrophil gelatinase-associated lipocalin (NGAL)	Dyga et al. [13]
Transferrin (TfR)	An and Xia [12]
Tissue inhibitor matrix metalloproteinase-1 (TIMP-1)	Qin et al. [27]
Urinary angiotensinogen (UAGT)	Ma et al. [17]
Urinary protein:Cr ratio (U-PCR)^a^	Ye et al. [23]

^a^Cr refers to creatinine

#### Urinary protein concentration

(i) Presence of nephritis: As expected, the 24h-UPRO was significantly increased in children with biopsy-proven IgAV-N (*n* = 694) compared to healthy controls (*n* = 400; *p* < 0.01). In a second paper, the urine Malb concentration was significantly increased in the IgAV-N group (*n* = 37) compared to both healthy controls and the IgAV-noN cohorts (*p* < 0.05) and the control group (*n* = 37) was not significantly different to the IgAV-noN patients (*n* = 34, *p* > 0.05) [16].
(ii) Severity of nephritis: Importantly, differences could be seen within the IgAV-N cohort when comparing histological grades I and IIa versus IIb, IIIa and IIIb (all *p* < 0.01). The AUC value was 0.77 for 24h-UPRO as a biomarker in distinguishing histology grades IIb, IIIa and IIIb. UPCR was also evaluated when assessing the severity of nephritis producing an AUC value of 0.73 [23]. Malb positively correlated with the grading of IgAV-N (*n* = 45, *p* < 0.05), with excellent AUC values for histological comparison (grade I vs. II AUC 0.95, 95% CI 0.87–1.00; grade II vs. III AUC 0.81, 95% CI 0.66–0.95; grade I vs. III AUC 0.98, 95% CI 0.94–1.00) [12].

#### Urinary β2-MG

(i) Presence of nephritis: One paper found that urine β2-MG was significantly increased in IgAV-N patients (*n* = 37) compared to both healthy controls (*n* = 37) and IgAV-noN (*n* = 34, *p* < 0.05) [16]. Qin et al. reported statistically significantly increased urinary concentration of β2-MG in children with IgAV-N (*n* = 66) compared to children with IgAV-noN (*n* = 68, *p* < 0.05) [20].
(ii) Severity of nephritis: Another paper (IgAV-N, *n* = 45) compared urinary β2-MG with the histological grades, grouped according to the ISKDC classification [10]. They found that urinary β2-MG was statistically significantly increased in all groups (*p* < 0.05) with no statistical difference between the histological classifications [12]. Zhang et al. explored urinary β2-MG in predicting irreversible kidney damage (defined as histological changes according to the ISKDC criteria) and reported a poor AUC at 0.49 (95% CI = 0.35–0.63, *p* = 0.89) [24].

#### Urinary KIM-1

(i) Presence of nephritis: This was reported as a potential biomarker in two studies. Dyga et al. found that KIM-1 was statistically significantly increased acutely in all IgAV patients (*n* = 29) when compared to the controls (*p* < 0.005) but there was no significant difference between IgAV-noN (*n* = 18) and IgAV-N (*n* = 11). Urinary KIM-1 concentrations decreased over time in IgAV-N and IgAV-noN [13]. Zhang et al. found the contrary, with mean urinary KIM-1 concentrations significantly increased in IgAV-N (*n* = 32) compared to IgAV-noN (*n* = 27, *p* < 0.05) and healthy controls (*n* = 16, *p* < 0.05). The AUC for KIM-1 in predicting nephritis was outstanding at 0.93 (95% CI = 0.88–0.99, *p* < 0.05) [24].
(ii) Severity of nephritis: A positive correlation between urinary KIM-1 levels and histological grade or total urine protein was found (*r* = 0.671, *p* < 0.01) [24]. Another paper found no statistical difference in distinguishing severity [13].

#### Urinary MCP-1

(i) Presence of nephritis: This was found to correlate with IgAV-N in two studies, reporting 447 children. Fuentes et al. reported a statistically significantly increased urinary MCP-1/Cr concentration in the IgAV-N cohort (*n* = 57) compared to healthy controls (*n* = 25) or IgAV-noN (*n* = 27, *p* < 0.01) [15]. Wang et al. also found urinary MCP-1 to be significantly increased in IgAV-N (*n* = 126) compared to healthy controls (*n* = 84, *p* < 0.01) and IgAV-noN (*n* = 135, *p* < 0.01). Urine MCP-1 concentrations increased in parallel with the degree of urinary protein concentration [21].
(ii) Severity of nephritis: One paper found that the AUC for MCP-1 predicting nephritis was excellent (AUC 0.83 95% CI = 0.73–0.92, *p* < 0.01) [15].

#### Urinary NAG

(i) Presence of nephritis: Zhang et al. also found increased urinary NAG concentration in IgAV-N (*n* = 32) compared to IgAV-noN (*n* = 27, *p* < 0.05). There was no difference between IgAV-noN (*n* = 27) and healthy controls (*n* = 16). The AUC for urinary NAG in distinguishing patients with nephritis was excellent (AUC 0.82 95% CI 0.72–0.92, *p* < 0.01) [24].
(ii) Severity of nephritis: An and Xia evaluated urinary NAG in biopsy-proven IgAV-N (*n* = 45). The concentrations correlated with increasing histological grade (*p* < 0.05) and the AUC in predicting the histological grades were excellent for grade I vs. II (AUC 0.84 95% CI 0.67–1.00), outstanding for grade I vs. III (AUC 0.96 95% CI 0.89–1.00); and acceptable for grade II vs. III (AUC 0.76 95% CI 0.59–0.93) [12].

#### Urinary angiotensinogen (UAGT)

(i) Presence of nephritis: Ma et al. compared IgAV-N (*n* = 14), IgAV-noN (*n* = 28) and healthy controls (*n* = 23). UAGT/Cr was significantly increased in IgAV-N compared to healthy controls and IgAV-noN (*p* < 0.05). This paper was unavailable in full text in English so limited data was extracted from the abstract only [17]. Mao et al. further subdivided patients with IgAV-N and described acute increase in UAGT in IgAV-N patients with a high urinary protein concentration (*n* = 13) compared to both IgAV-noN (*n* = 51) and IgAV-N with only haematuria (*n* = 43, *p* < 0.01). This finding remained even during the convalescent phase where UAGT concentrations remained increased in the IgAV-N with a high urinary protein concentration compared to the IgAV-noN (*p* < 0.01) and the IgAV-N with haematuria (*p* < 0.01). The difference in concentration during the convalescent phase between the IgAV-noN and IgAV-N with haematuria was not significant [18].
(ii) Severity of nephritis: No studies assessed UAGT to determine the severity of nephritis.

## Discussion

This systematic review aimed to identify current clinical and potential pre-clinical urine biomarkers associated with the presence of nephritis and its severity in children with IgAV-N. Using a predetermined systematic evaluation, we have reported a cohort of 2446 children, including 1685 children with IgAV, using data from 13 papers. These data identified 23 potential biomarkers described in the literature including the clinical biomarker of urinary protein concentration and 5 pre-clinical urine biomarkers that had been evaluated by more than one study. Of these pre-clinical biomarkers, 4 demonstrated promising association with IgAV nephritis: KIM-1, MCP-1, NAG and UAGT [13, 15, 17, 18, 22, 24]. One urine biomarker, β2-MG, although frequently studied, did not perform well [12, 16, 24]. A further 18 markers were less frequently reported but were summarised as they may have potential future utility in this disease and provide important insight into the underlying pathophysiology.

The clinical biomarker that performed best at assessing the severity of nephritis was urinary albumin concentration with excellent AUC values (AUC 0.81–0.98) in determining the grade of histological inflammation in IgAV-N. The pre-clinical biomarkers, KIM-1, MCP-1, NAG and UAGT, demonstrate promise for their association with either the presence or severity of nephritis, and their relative advantages and disadvantages are summarised in Table 3.

**Table 3:** A table comparing the clinical and pre-clinical biomarkers, their AUC values and their advantages and disadvantages

Biomarker		AUC values	Region of kidney predominantly released from	Advantages	Disadvantages
Urinary protein concentration	Urinary albumin concentration	0.81–0.98	Glomerulus	• Established marker of disease • Available in clinical laboratories • Associated with prediction of severity of nephritis	• Only present when damage has already occurred as it is a sign of kidney damage • Albuminuria superior to proteinuria • 24-UPRO rarely performed in practice
	24-h urinary protein (24h-UPRO) or protein:creatinine ratio (PCR)	0.73–0.77	Glomerulus		
Kidney injury molecule-1 (KIM-1)		0.93	Tubulointerstitial	• Not expressed in other organs so very specific • Outstanding AUC • Has been suggested to correlate with IgAV-N and IgA nephropathy in the adult population where correlation with the degree of tubulointerstitial injury was also reported [31, 32]	• May only be released due to downstream result of glomerular damage • One paper found no clear relationship • Not yet an established marker of disease • Not reported to correlate with histology
Monocyte chemoattractant protein-1 (MCP-1)		0.83	Glomerular	• Reported to provide early identification of nephritis and predict histology in two papers • Associated with histology • Previously found to be associated with IgA nephropathy and lupus nephritis in adult populations	• Not yet an established marker of disease
N-Acetyl-beta-glucosaminidase (NAG)		0.82	Tubular	• Early identification of nephritis and predictive potential, able to correlate with histology	• Few previous studies on IgA-mediated diseases • Not yet an established marker of disease • May only be released due to downstream result of glomerular damage
Urinary angiotensinogen (UAGT)		n/a	Glomerular and/or tubular	• May imply novel pathophysiology not previously studied	• No AUC value to compare • Not yet an established marker of disease • If tubular involvement, may only be released due to downstream result of glomerular damage

In addition to highlighting promising biomarkers, this study provides insight into key biological pathways in IgAV-N. The fact that many of the most promising biomarkers arise as a result of tubulointerstitial inflammation is an extremely interesting finding as IgAV-N is traditionally considered solely a glomerulonephritis. Examples of these markers are KIM-1 and NAG. KIM-1 is a type 1 transmembrane protein that is absent in the normal kidney, upregulated in tubular injury and not expressed in other organs [33]. It is a recognised biomarker in acute tubular necrosis and allograft nephropathy where it has been found to correlate with the degree of tubulointerstitial insult [34–36]; however, it has not yet been reported in the histology for IgAV-N. This review included one small study that found no clear relationship between KIM-1 concentration and IgAV-N but it did demonstrate a reduction over time suggesting some relationship with disease activity [13]. A larger study by Zhang et al. reported an outstanding AUC (0.93) for KIM-1 in its ability to identify IgAV-N [37, 38]. The lysosomal enzyme NAG is found in many body tissues, but it is found in particularly high concentrations in the proximal kidney tubular cells. NAG may be released into the urine via exocytosis or, more commonly, during kidney injury causing proximal tubule leakage [39]. Urinary NAG has been described in patients with acute kidney injury and more recently in diabetic nephropathy; however, there are few studies in IgA-mediated kidney diseases [40–42]. Our review found urinary NAG as a promising biomarker, able to distinguish patients with IgAV-N from those without nephritis [37] and accurately correlate with the degree of histopathology in IgAV-N [12]. This suggests that tubular inflammation may play a larger role than previously thought and warrants further evaluation. Tubular markers may be evident due to tubular damage leading to urinary release of these proteins as a downstream result of glomerular damage or from direct tubular involvement. Tubulointerstitial components have recently been added to proposed histological scoring classification systems for IgAV-N due to their better correlation with clinical outcomes. This supports the finding that the tubulointerstitial region may be of importance in this disease [43].

Nephritis is the main long-term complication of IgAV and there is currently no way to predict and identify which children may get irreversible kidney damage from the outset, thus all children are committed to a period of at least 6 months of monitoring. A better understanding of the underlying biology represented by urine biomarkers may allow identification of children who are at low or high risk of disease progression allowing monitoring stratification from the outset. Further studies are required to demonstrate whether pre-clinical markers are superior to current clinical biomarkers in terms of their ability to earlier detect nephritis or predict severity.

Limitations of this study include some studies being small and the heterogeneous nature of the papers regarding descriptive statistics, definition of nephritis, and type of sampling, methodologies, outcomes and data presentation made comparisons challenging. This review has identified the need for standardisation of biomarker evaluation in this disease to allow systematic comparison in the future. Some papers had missing data and one was only available in abstract form in English. The majority of these studies were cross sectional in design, so future longitudinal studies are needed to evaluate how the biomarkers change with the course of disease. Finally, most of the papers included in our review were from China and the relevance of ethnic variation of the expression of urinary biomarkers is currently unknown.

## Conclusion

Overall, this study suggests that there are promising urine biomarkers for IgAV-N and some of these also originate from the tubulointerstitial region suggesting a pathophysiological role. In order to assess their true potential as adjuncts to clinical practice, long-term evaluation of these urine biomarkers is needed.

## References

[1] Gardner-Medwin JM, Dolezalova P, Cummins C, Southwood TR (2002). Incidence of Henoch-Schönlein purpura, Kawasaki disease, and rare vasculitides in children of different ethnic origins. Lancet.

[2] Jennette JC, Falk RJ, Bacon PA, Basu N, Cid MC, Ferrario F, Flores-Suarez LF, Gross WL, Guillevin L, Hagen EC, Hoffman GS, Jayne DR, Kallenberg CGM, Lamprecht P, Langford CA, Luqmani RA, Mahr AD, Matteson EL, Merkel PA, Ozen S, Pusey CD, Rasmussen N, Rees AJ, Scott DGI, Specks U, Stone JH, Takahashi K, Watts RA (2013). 2012 revised international Chapel Hill consensus conference nomenclature of vasculitides. Arthritis Rheum.

[3] Heineke MH, Ballering AV, Jamin A, Ben Mkaddem S, Monteiro RC, Van Egmond M (2017). New insights in the pathogenesis of immunoglobulin A vasculitis (Henoch-Schönlein purpura). Autoimmun Rev.

[4] Blanco R, Martínez-Taboada VM, Rodríguez-Valverde V, García-Fuentes M, González-Gay MA (1997). Henoch-Schönlein purpura in adulthood and childhood: two different expressions of the same syndrome. Arthritis Rheum.

[5] Nong BR, Huang YF, Chuang CM, Liu CC, Hsieh KS (2007). Fifteen-year experience of children with Henoch-Schönlein purpura in southern Taiwan, 1991–2005. J Microbiol Immunol Infect.

[6] Jauhola O, Ronkainen J, Koskimies O, Ala-Houhala M, Arikoski P, Hölttä T, Jahnukainen T, Rajantie J, Ormälä T, Nuutinen M (2010). Clinical course of extrarenal symptoms in Henoch-Schonlein purpura: a 6-month prospective study. Arch Dis Child.

[7] Oni L, Sampath S (2019). Childhood IgA vasculitis (Henoch Schonlein purpura)-advances and knowledge gaps. Front Pediatr.

[8] Narchi H (2005). Risk of long term renal impairment and duration of follow up recommended for Henoch-Schonlein purpura with normal or minimal urinary findings: a systematic review. Arch Dis Child.

[9] Ozen S, Pistorio A, Iusan SM, Bakkaloglu A, Herlin T, Brik R, Buoncompagni A, Lazar C, Bilge I, Uziel Y, Rigante D, Cantarini L, Hilario MO, Silva CA, Alegria M, Norambuena X, Belot A, Berkun Y, Estrella AI, Olivieri AN, Alpigiani MG, Rumba I, Sztajnbok F, Tambic-Bukovac L, Breda L, Al-Mayouf S, Mihaylova D, Chasnyk V, Sengler C, Klein-Gitelman M, Djeddi D, Nuno L, Pruunsild C, Brunner J, Kondi A, Pagava K, Pederzoli S, Martini A, Ruperto N (2010). EULAR/PRINTO/PRES criteria for Henoch-Schönlein purpura, childhood polyarteritis nodosa, childhood Wegener granulomatosis and childhood Takayasu arteritis: Ankara 2008. Part II: final classification criteria. Ann Rheum Dis.

[10] Huang X, Ma L, Ren P, Wang H, Chen L, Han H, Chen J, Han F (2019). Updated Oxford classification and the international study of kidney disease in children classification: application in predicting outcome of Henoch-Schönlein purpura nephritis. Diagn Pathol.

[11] Downes MJ, Brennan ML, Williams HC, Dean RS (2016). Development of a critical appraisal tool to assess the quality of cross-sectional studies (AXIS). BMJ Open.

[12] An JK, Xia D (2018). Diagnostic performance of urinary proteins as biomarkers in evaluating Henoch Schonlein purpura nephritis. Clin Exp Med.

[13] Dyga K, Machura E, Świętochowska E, Szczepańska M (2020). Analysis of the association between kidney injury biomarkers concentration and nephritis in immunoglobulin A vasculitis: a pediatric cohort study. Int J Rheum Dis.

[14] Fang X, Wu HY, Lu M, Cao Y, Wang R, Wang MQ, Gao CL, Xia ZK (2020). Urinary proteomics of Henoch-Schonlein purpura nephritis in children using liquid chromatography-tandem mass spectrometry. Clin Proteomics.

[15] Fuentes Y, Hernández AM, García-Roca P, Valverde S, Velásquez-Jones LF, Sosa G, Duarte-Durán UO, Ortíz L, Maldonado R, Faugier E, Ramón-García G, Medeiros M (2014). Urinary MCP-1/creatinine in Henoch-Schönlein purpura and its relationship with nephritis. Pediatr Nephrol.

[16] Ge W, Wang H-L, Sun R-P (2014). Pentraxin 3 as a novel early biomarker for the prediction of Henoch-Schonlein purpura nephritis in children. Eur J Pediatr.

[17] Ma YF, Li YF, Guo GM, Zhu YJ, Gong YL, Dong Y (2020). Changes of new urinary biomarkers in children with Henoch-Schonlein purpura nephritis. J Shanghai Jiaotong Univ Med Sci.

[18] Mao YN, Liu W, Li YG, Jia GC, Zhang Z, Guan YJ, Zhou XF, Liu YF (2012). Urinary angiotensinogen levels in relation to renal involvement of Henoch-Schonlein purpura in children. J Nephrol.

[19] Pillebout E, Jamin A, Ayari H, Housset P, Pierre M, Sauvaget V, Viglietti D, Deschenes G, Monteiro RC, Berthelot L (2017). Biomarkers of IgA vasculitis nephritis in children. PLoS One.

[20] Qin Y-H, Zhou T-B, Lei F-Y, Huang W-F, Zhao Y-J, Lin F-Q, Su L-N (2011). Cut-off values for serum matrix metalloproteinase-9: is there a threshold to predict renal involvement for Henoch-Schonlein purpura in children?. Nephrology (Carlton).

[21] Wang J, Ying Q, Zhong S, Chen Y, Di Y, Dai X, Zheng J, Shen M (2018). Elevated urinary monocyte chemoattractant protein-1 levels in children with Henoch-Schonlein purpura nephritis. Pediatr Neonatol.

[22] Wang J, Ying Q, Zhong S, Chen Y, Di Y, Dai X, Zheng J, Shen M (2017). Elevated urinary monocyte chemoattractant protein-1 levels in children with Henoch-Schonlein purpura nephritis. Pediatr Neonatol.

[23] Ye Q, Shang SQ, Liu AM, Zhang T, Shen HQ, Chen XJ, Mao JH (2015). 24h urinary protein levels and urine protein/creatinine ratios could probably forecast the pathological classification of HSPN. PLoS One.

[24] Zhang J, Zeng H, Wang N, Tian X, Dou W, Shi P (2015). Beneficial effects of creatine phosphate sodium for the treatment of Henoch-Schönlein purpura in patients with early renal damage detected using urinary kidney injury molecule-1 levels. Eur J Pediatr.

[25] Califf RM (2018). Biomarker definitions and their applications. Exp Biol Med (Maywood).

[26] Mandrekar JN (2010). Receiver operating characteristic curve in diagnostic test assessment. J Thorac Oncol.

[27] Qin YH, Zhou TB, Lei FY, Huang WF, Zhao YJ, Lin FQ, Su LN (2011). Cut-off values for serum matrix metalloproteinase-9: is there a threshold to predict renal involvement for Henoch-Schonlein purpura in children?. J Nephrol.

[28] Ge W, Wang HL, Sun RP (2014). Pentraxin 3 as a novel early biomarker for the prediction of Henoch-Schönlein purpura nephritis in children. Eur J Pediatr.

[29] Wang JP, Li YY, Chen YL, Dai XH, Di YZ, Shen MJ, Ying QQ, Fu SW, Li YJ (2017). Urinary macrophage migration inhibitory factor as a noninvasive biomarker in pediatric Henoch-Schonlein purpura nephritis. J Clin Rheumatol.

[30] Wang J, Li Y, Chen Y, Dai X, Di Y, Shen M, Ying Q, Fu S, Li Y (2017). Urinary macrophage migration inhibitory factor as a noninvasive biomarker in pediatric Henoch-Schonlein purpura nephritis. J Clin Rheumatol.

[31] Zhang Y, Li A, Wen J, Zhen J, Hao Q, Zhang Y, Hu Z, Xiao X (2017). Kidney injury molecule-1 level is associated with the severity of renal interstitial injury and prognosis in adult Henoch-Schönlein purpura nephritis. Arch Med Res.

[32] Xu P-C, Zhang J-J, Chen M, Lv J-C, Liu G, Zou W-Z, Zhang H, Zhao M-H (2011). Urinary kidney injury molecule-1 in patients with IgA nephropathy is closely associated with disease severity. Nephrol Dial Transplant.

[33] Song J, Yu J, Prayogo GW, Cao W, Wu Y, Jia Z, Zhang A (2019). Understanding kidney injury molecule 1: a novel immune factor in kidney pathophysiology. Am J Transl Res.

[34] Han WK, Bailly V, Abichandani R, Thadhani R, Bonventre JV (2002). Kidney injury molecule-1 (KIM-1): a novel biomarker for human renal proximal tubule injury. Kidney Int.

[35] Edelstein CL (2017) Chapter six - biomarkers in acute kidney injury. In: Edelstein CL (ed) Biomarkers of kidney disease (Second Edition). Academic Press, pp 241-315

[36] Waanders F, van Timmeren MM, Stegeman CA, Bakker SJL, van Goor H (2010). Kidney injury molecule-1 in renal disease. J Pathol.

[37] Zhang J, Zeng H, Wang N, Tian X, Dou W, Shi P (2016). Beneficial effects of creatine phosphate sodium for the treatment of Henoch-Schönlein purpura in patients with early renal damage detected using urinary kidney injury molecule-1 levels. Eur J Pediatr.

[38] Liu F, Wang C, Wang R, Wang W, Li M (2018). Henoch-Schonlein purpura nephritis with renal interstitial lesions. Open Med (Wars).

[39] Wen X, Kellum JA, Vincent J-L, Hall JB (2012). N-Acetyl-beta-D-glucosaminidase (NAG). Intensive care med.

[40] Vaidya VS, Ferguson MA, Bonventre JV (2008). Biomarkers of acute kidney injury. Annu Rev Pharmacol Toxicol.

[41] Sheira G, Noreldin N, Tamer A, Saad M (2015). Urinary biomarker N-acetyl-β-D-glucosaminidase can predict severity of renal damage in diabetic nephropathy. Diabetes Metab Syndr.

[42] Bazzi C, Petrini C, Rizza V, Arrigo G, Napodano P, Paparella M, D'Amico G (2002). Urinary N-acetyl-beta-glucosaminidase excretion is a marker of tubular cell dysfunction and a predictor of outcome in primary glomerulonephritis. Nephrol Dial Transplant.

[43] Koskela M, Ylinen E, Ukonmaanaho EM, Autio-Harmainen H, Heikkilä P, Lohi J, Jauhola O, Ronkainen J, Jahnukainen T, Nuutinen M (2017). The ISKDC classification and a new semiquantitative classification for predicting outcomes of Henoch-Schönlein purpura nephritis. Pediatr Nephrol.

